# The Ocular Surface and the Coronavirus Disease 2019: Does a Dual ‘Ocular Route’ Exist?

**DOI:** 10.3390/jcm9051269

**Published:** 2020-04-28

**Authors:** Pietro Emanuele Napoli, Matteo Nioi, Ernesto d’Aloja, Maurizio Fossarello

**Affiliations:** 1 Department of Surgical Science, University of Cagliari, Eye Clinic, via Ospedale 46, 09124 Cagliari, Italy; 2 Department of Clinical Sciences and Public Health, University of Cagliari, Forensic Medicine Unit, 09124 Cagliari, Italy; 3 Clinica Oculistica, San Giovanni di Dio Hospital, Azienda Ospedaliera Universitaria di Cagliari, 09124 Cagliari, Italy

**Keywords:** COVID-19, coronavirus, SARS-CoV-2, ACE-2 receptor, eye, conjunctivitis, ocular surface, cornea, transmission, dual ocular route

## Abstract

Coronavirus disease 2019 (COVID-19) is an important health problem that was defined as a pandemic by the World Health Organization on 11 March 2020. Although great concern has been expressed about COVID-19 infection acquired through ocular transmission, its underlying mechanism has not currently been clarified. In the current work, we analyzed and elucidated the two main elements that should be taken into account to understand the “ocular route”, both from a clinical and molecular point of view. They are represented by the dynamism of the ocular surface system (e.g., the tear film turnover) and the distribution of ACE2 receptors and TMPRSS2 protein. Although it seems, at the moment, that there is a low risk of coronavirus spreading through tears, it may survive for a long time or replicate in the conjunctiva, even in absence of conjunctivitis signs, indicating that eye protection (e.g., protective goggles alone or in association with face shield) is advisable to prevent contamination from external droplets and aerosol.

## 1. Introduction

Coronavirus disease 2019 (COVID-19) is an important health problem that was defined as a pandemic by the World Health Organization (WHO) on 11 March 2020 [1,2]. This global epidemic is determined by a novel betacoronavirus named “severe acute respiratory syndrome coronavirus 2” (SARS-CoV-2), whose ease of interpersonal transmission is one of the crucial factors in the spread of the disease [3]. SARS-CoV-2 belongs to the same betacoronavirus family as SARS-CoV and Middle East respiratory syndrome-related coronavirus (MERS-CoV), the other two viruses that caused outbreaks in the past two decades.

The main routes of transmission of the infection are considered as follows: airborne dissemination (nose/throat or ‘respiratory route’ through aerosols, dust or liquids), direct or indirect contact (e.g., face/eye touching in case of conjunctivitis), and oral-fecal.

## 2. Ocular Surface Findings in Case of COVID-19 and Controversial Issues

SARS-CoV-2 conjunctivitis has been described as a mild follicular conjunctivitis otherwise indistinguishable from other viral causes, and be potentially transmitted by aerosol contact with conjunctiva [4]. Other features of ocular surface involvement include unilateral or bilateral bulbar conjunctiva hyperemia alone or in association with chemosis, follicular reaction of the palpebral conjunctiva, watery discharge, epiphora, and mild eyelid edema. There have been no reports of COVID-19 patients experiencing blurred vision.

The prevalence of conjunctivitis in patients with COVID-19 is controversial. Although it has been reported that only 0.9% developed signs of conjunctivitis [5], other report indicates that up to 31.6% of hospitalized patients had conjunctivitis [6]. 

However, in the latter study, only about 5% of patients with positive findings for COVID-19 on reverse transcriptase-polymerase chain reaction (RT-PCR) from nasopharyngeal swabs showed a positive conjunctival swab. In no cases was a positive conjunctival swab associated with a negative nasopharyngeal swab. Moreover, only one patient out of 38 presented with conjunctivitis as the first symptom. Patients with ocular symptoms were more likely to have higher white blood cell and neutrophil counts, as well as higher levels of procalcitonin, C-reactive protein, and lactate dehydrogenase than patients without ocular symptoms. In one patient, RT-PCR assay demonstrated the presence of viral RNA in conjunctival specimen 13 days after onset. The conjunctival swab specimens remained positive for SARS-CoV-2 on 14 and 17 days after onset. On day 19, RT-PCR result was negative for SARS-CoV-2 [4].

In another report, one hospitalized patient showed SARS-CoV-2 positive conjunctival swabs up to 21 days from symptom onset, a few days after the virus was undetectable in nasal swabs. Five days after, the virus resulted undetectable in the conjunctival swab, it was detected again at day 27, suggesting sustained replication of the virus in conjunctiva [7].

## 3. Ocular Transmission and the ACE2 Receptors in the Ocular Surface

Although great concern has been expressed about COVID-19 infection acquired through ocular transmission, its underlying mechanism has not yet been clarified [8].

SARS-CoV-2, like the others betacoronaviruses [9], entry into target cells by the binding of the viral Spike (S) protein to a specific cell-surface receptor, called angiotensin converting enzyme 2 (ACE2), followed by its priming by host cell transmembrane protease, serine 2 (TMPRSS2) [10]. Although much is still to be learned about the factors involved in SARS-CoV-2 infection of human cells, ACE2 and TMPRSS2 are currently believed to represent the major players during cell entry [10,11].

The presence of ACE2, which normally helps regulate blood pressure, is widespread throughout the body, and the eye is not excluded [12,13]. The human ocular globe has its own intraocular renin-angiotensin system, which is present not only on the surface of the eye (e.g., conjunctiva and cornea), but also inside the eye (trabecular meshwork, aqueous humor, iris, ciliary body, non-pigmented ciliary epithelium, and retina) [14]. TMPRSS2 is also highly expressed in various tissues, including cornea limbal stem cells, substantiating that in the cornea ACE2 and TMPRSS2 are coexpressed in the same cell [15].

Accordingly, the two main elements that should be taken into account to understand the “ocular route”, from a clinical and molecular point of view, are the *dynamism* of the ocular surface system [16] and the distribution of *ACE2 receptors and TMPRSS2 protein*.

In the first place, the dynamism of the tear film is the factor supporting SARS-CoV-2 to pass from the infected ocular surface to the respiratory and digestive tract through the *lacrimal canaliculi* (that drain tears from the eye surface into the nasal cavity), regardless of a more or less significant presence of ACE2 receptors on the cornea and conjunctiva [17,18]. The opposite passage of viruses from nasal mucosa to conjunctiva seems unlikely, but cannot be excluded, as well as haematogenous infection of the lacrimal gland.

Clearly, the lacrimal drainage from conjunctival sac into the nasal cavity is not or only partially operating in people with dry eye, for which lacrimal substitutes are highly recommended. On the other hand, the obstruction (complete or partial) of lacrimal drainage pathways may play a role in retaining the coronavirus on the ocular surface regardless of its presence in the nasal cavity, thus promoting periocular/face skin contamination by means of epiphora (i.e., the excessive watering of the eye).

Secondly, the presence of the ACE2 receptors and TMPRSS2 protein on the corneal limbal stem cells [15] may theoretically allow the betacoronavirus to cross the ocular surface, and then spread from the eye to other parts of the body through the blood stream and/or the nervous system (ophthalmic branch of trigeminal nerve) [19]. Although there are no evidences at the moment that COVID-19 virus, in humans, can enter inside the eye or spread to the brain through corneal nerves, in some animal models (feline and murine), betacoronaviruses can cause several ocular affections (e.g., conjunctivitis, uveitis, retinitis and optic neuritis), thus suggesting that they are able, in some mammals, to penetrate inside the ocular globe [20].

Overall, preliminary studies reveal abundant gene expression of ACE2 receptor in the human conjunctiva and cornea [18] together with TMPRSS2 protein [15]. The coexpression in the corneal limbal cells of ACE2 and TMPRSS2 [15] substantiates the high affinity of this tissue for SARS-CoV-2 and its presence in tears. Therefore, in eyes with normal tearing (typically in young subjects) the dispersion of the viral load in the tear film may convey the virus into the respiratory or digestive tract, while in a dry eye the corneal and conjunctival epithelium may more easily retain the virus (for example in elderly people, where this disorder is very frequent), favoring a reactive/infective keratoconjunctivitis.

## 4. Discussion

A number of experimental and clinical evidences support the hypothesis that SARS-CoV-2 can be found in tears, and that it can therefore be received and transmitted through this route. Although it seems at the moment that the virus cannot be detected in the conjunctival sac of infected patients without conjunctivitis [21], and that there is a low risk of coronavirus spreading through tears [22], nevertheless SARS-CoV-2 may survive for a long time or replicate in the conjunctiva, after conjunctivitis signs vanished. Therefore, eye protection (protective goggles alone or in association with face shield) are necessary to prevent eye exposure to contaminated droplets and bioaerosol emitted by patients not only by cough and sneeze, but also by breathing [23,24,25]. Obviously, eye care providers and technicians may be more susceptible to infection due to the nature and proximity of the ophthalmic examination [26]. Eye care providers are encouraged to use slit lamp breath shields and should counsel patients to speak as little as possible when sitting at the slit lamp to reduce the risk of virus transmission. Disinfection and sterilization practices should be employed for shared clinic equipment such as tonometers, trial frames, pinhole occluders, near vision cards, B-scan probes, and contact lenses for laser procedures. Clearly, the use of disposable material (e.g., tonometer tips or gloves), as far as possible, would also be desirable during eye examinations (together with all other recommended personal protective equipment).

However, eye protection is recommended not only for all health care workers, but also for various categories of people at risk (e.g., immunocompromised patients located in small rooms with poor ventilation, or older people living in close communities, such as health care residences) [27,28,29], and for individuals with ocular surface diseases (e.g., dry eye) or at risk of corneal ulcer (e.g., contact lens wearers). In this respect, personal eyeglasses and contact lenses do not qualify as personal protective equipment, although contact lenses remain a perfectly acceptable form of vision correction during the coronavirus pandemic, as long as people practice good hand hygiene and follow appropriate wear-and-care directions [20].

In conclusion, scientific literature on human ocular SARS-CoV-2 infection is growing rapidly, and this will help to clarify the role played by specific ocular routes in the transmission of COVID-19.
